# Effects of meridian sinew tuina after identifying the treatment area under ultrasound localization combined with greater and third occipital nerve injections in cervicogenic headache: a randomized controlled trial protocol

**DOI:** 10.3389/fneur.2024.1439922

**Published:** 2024-09-02

**Authors:** Qinghua Huang, Yuxuan Li, Lijun Ou, Liyu Gong, Jianlin Quan, Jiayi Kuang, Sijie Tao, Shiyao Zhang

**Affiliations:** ^1^Guangxi University of Chinese Medicine, Nanning, Guangxi, China; ^2^Department of Tuina, Guilin Municipal Hospital of Traditional Chinese Medicine, Guilin, Guangxi, China; ^3^Guangzhou University of Chinese Medicine, Guangzhou, China

**Keywords:** cervicogenic headache, meridian sinew tuina, ultrasound localization, greater occipital nerve, third occipital nerve, medical infrared thermography

## Abstract

**Introduction:**

Cervicogenic headache (CEH) is a secondary headache characterized by chronic, unilateral headache. Ultrasound-guided injections of the greater occipital nerve (GON) and the third occipital nerve (TON) are effective in the treatment of CEH, as is meridian sinew tuina for the treatment of CEH, but the evidence of clinical efficacy of combining these two therapies is valid. Therefore, we have designed a randomized controlled trial with the aim of investigating the efficacy and safety of ultrasound localization meridian sinew tuina combined with GON and TON injections for the treatment of CEH.

**Methods and analysis:**

In this study, we enroll 60 patients experiencing CEH. The control group receives ultrasound-guided injections of GON and TON. The intervention group is treated with ultrasound localization meridian sinew tuina combined with the injection of GON and TON. Meridian sinew tuina is performed once a day for 30 min for 3 days. The primary observational index includes the Short-Form of McGill Pain Questionnaire (SF-MPQ). The Secondary outcomes include Cervical Range of Motion (ROM) and Medical Infrared Thermography (MIT). MIT is used to measure the change in skin temperature in the area of the patient’s meridian sinew tuina treatment of GON and TON before and after the intervention. There are 5 time points assessed as baseline, day 3, day 15, day 30, and day 60.

**Discussion:**

This study proposes to combine ultrasound-guided injections of GON and TON for the treatment of CEH after identifying the treatment area of meridian sinew tuina under ultrasound localization. Meanwhile, MIT is utilized to provide objective evidence of the efficacy of CEH.

**Clinical trial registration:**

ChiCTR2300076128.

## Introduction

Cervicogenic headache (CEH) is a syndrome manifesting as chronic, unilateral headaches originating from organic or functional abnormalities in the cervical spine or its associated soft tissues (1). Epidemiological evidence suggests the prevalence of CEH ranges from 2.2 to 4.1%, with figures potentially rising to 15–20% in individuals experiencing chronic headaches (2–4). The pathogenesis of CEH is primarily linked to lesions or inflammatory irritations of the cervical structures innervated by the higher cervical nerves, leading to pain that may extend to the occipital, cervical, parietal, frontal, and periorbital areas (5, 6). The greater and third occipital nerves (GON and TON) are notably implicated in the onset of CEH, with involvement of the cervical nerve being a prevalent cause (7).

Current physical therapies for CEH include cold packs, spinal manipulation, and posture enhancement (8, 9). Nonetheless, pharmacological treatments such as nonsteroidal anti-inflammatory drugs (NSAIDs) and opioids offer only temporary relief and are associated with significant adverse effects (10, 11). Although radiofrequency ablation has effectively reduced CEH pain symptoms, it carries potential adverse effects, including sensory dullness and numbness, reported in 12–13% of cases (12, 13). This underscores the urgent need for new, safer, and more productive therapeutic options to improve the management of CEH.

Meridian sinew tuina, deriving from the Meridian Sinew theory and tuina therapy elucidated in the Emperor’s Internal Canon, is deeply rooted in traditional Chinese medical practices. Meridian Sinews (Jingjin), considered extensions of the twelve regular meridians, predominantly connect tendons, muscles, and joints. The foundational concepts of Meridian Sinew theory are detailed in the “Meridian Sinew” chapter of the Spiritual Pivot (Ling Shu) and its subsequent commentaries. This theory’s therapeutic potential for cervicogenic headache (CEH) has been explored, revealing a notable correlation between CEH and disturbances in the foot-sun meridian sinew, particularly given its extensive distribution in the head and neck area (14, 15). This distribution aligns with the regions innervated by the greater and third occipital nerves, correlating with the primary pain sites in CEH (16).

The anatomical structures associated with the foot-sun meridian sinew, ranging from superficial to deep, include the trapezius, splenius capitis, semispinalis capitis, rectus capitis posterior major, and Obliquus capitis inferior (17). Through the application of meridian sinew tuina, a manipulative therapy grounded in traditional Chinese medicine, there is potential to alleviate the compression on high cervical nerves, improve local blood circulation, reduce aseptic inflammation, and thus mitigate pain while relaxing stiff soft tissues in CEH patients (18). Clinical evidence suggests that meridian sinew tuina is a safe and effective treatment modality for CEH (19). Leveraging meridian sinew theory as a framework, Legge et al. reported favorable outcomes in managing chronic pain conditions (20).

However, the physiological mechanisms underpinning the Efficacy of meridian sinew tuina for CEH remain under-researched. Previous animal studies have shown that massage simulation can improve pain behavior in mice, an effect attributed to reduced peripheral inflammation. This reduction was mediated by the mechanosensitive channel protein Piezo and senescence-related pathways (21, 22). More recently, tuina manipulation in a neuropathic pain model was found to have an analgesic effect, potentially linked to the modulation of inflammation by noncoding RNA (23).

In 2019, the Chinese Society of Pain of the Chinese Medical Association published the clinical practice consensus titled “Clinical Diagnosis and Treatment of Cervicogenic Headache: A Consensus of Chinese Pain Specialists.” This consensus endorses the use of ultrasound-guided blocks of the greater and/or third occipital nerve (GON and/or TON) as a principal diagnostic and therapeutic approach for cervicogenic headache (CEH) (6). Traditionally, the technique for administering GON injections relied on superficial bony anatomical landmarks to infiltrate local anesthetics and corticosteroids around the nerves at the superior cervical line level. However, this approach risks inadvertently anesthetizing adjacent structures or intravascular injections into structures such as the occipital arteries due to imprecision in needle placement (24).

Ultrasound-guided techniques have revolutionized this procedure by allowing real-time visualization of the needle’s trajectory, thereby ensuring the precise delivery of an optimal dose of compounded betamethasone directly to the affected area. This method significantly reduces the localized inflammatory response, promptly relieving or eliminating pain. Additionally, ultrasound guidance provides clear visualization of the dispersion of the injected solution, reducing the likelihood of adverse effects (7, 25). Clinical evidence supports that ultrasound-guided interventions for GON and TON injections effectively alleviate CEH symptoms and improve patient quality of life (26). Given the critical nature of neck structures, this study will eschew the use of anesthetic agents to avoid the risk of severe complications.

This study introduces an innovative ultrasound localization technique for guiding meridian sinew tuina therapy in treating cervicogenic headache (CEH), followed by targeted injections into the greater and third occipital nerves. This method, devoid of radiation, offers a precision advantage over traditional body palpation and C-arm guided localization, allowing for the accurate release of the entrapped occipital nerves and adjacent soft tissues. Preliminary clinical trials have demonstrated that this integrative approach significantly alleviates pain symptoms in CEH patients.

Building on this foundation, our randomized controlled trial aims to investigate novel therapeutic strategies for CEH. We aim to assess the impact of combining ultrasound-localized meridian massage with injections into the greater and third occipital nerves. This approach will be evaluated based on its effectiveness in improving pain score indices, headache visual analog scores, pain intensity, and cervical spine mobility in CEH patients. This research seeks to contribute to developing more effective, precision-based treatments for those suffering from CEH.

## Methods and design

### Trial design

This study is designed as a single-center, randomized, parallel-controlled trial. Participants will be randomly allocated to one of two groups: the control group, which will receive ultrasound-guided injections into the greater and third occipital nerves (GON and TON), or the intervention group, which will undergo ultrasound-localized meridian sinew tuina in conjunction with GON and TON injections, maintaining a 1:1 ratio. The effectiveness of these treatments will be assessed at five distinct time points: at baseline, 3 days post-treatment commencement, 15 days post-treatment conclusion, and 30 and 60 days following the end of treatment.

**Figure 1:**
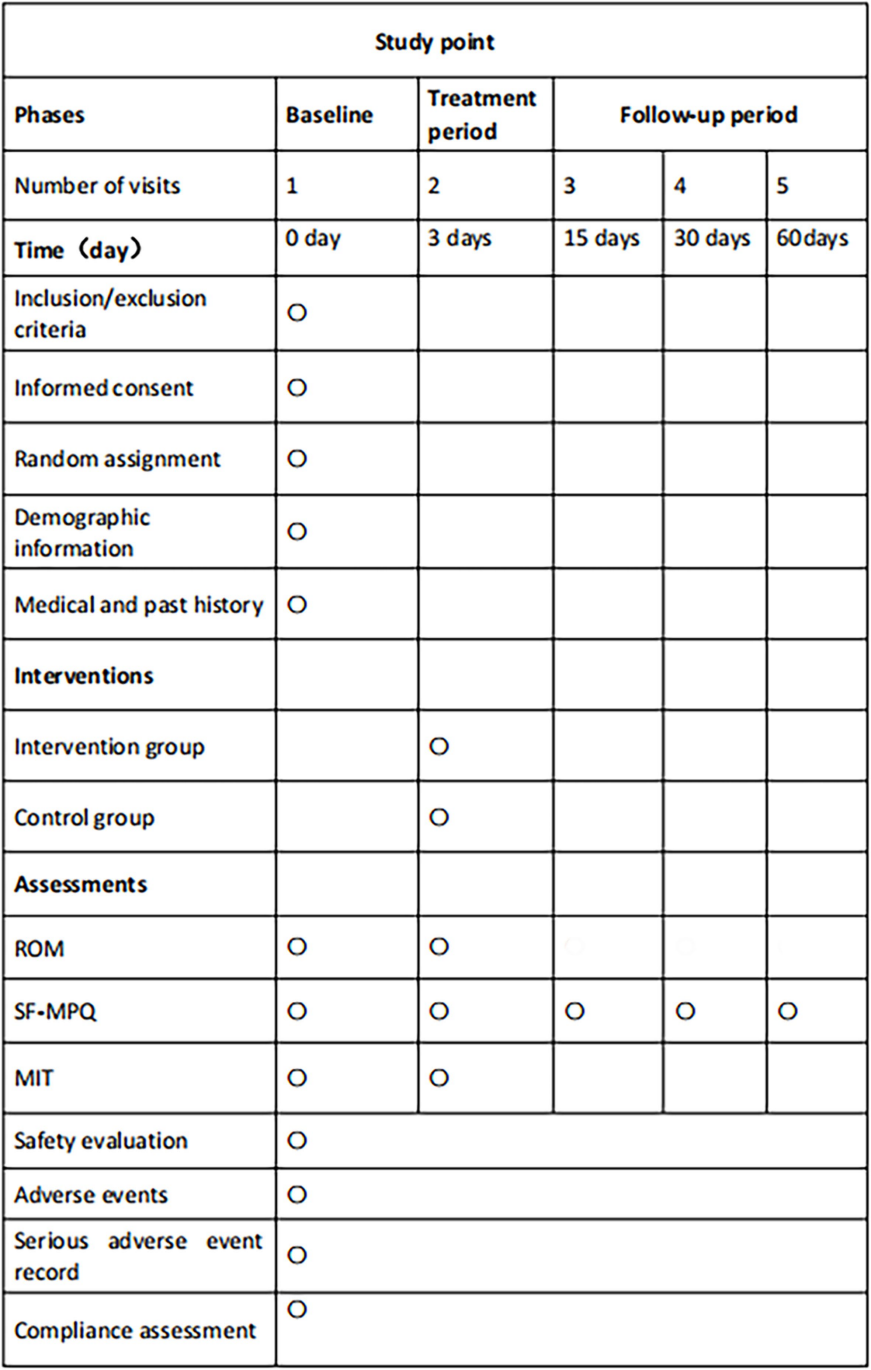
SPIRIT schedule. This figure outlines the phases, timing, interventions, and assessments planned throughout the trial. Key abbreviations include ROM (range of motion), SF-MPQ (Short-Form McGill Pain Questionnaire), and MIT (medical infrared thermography), with ○ denoting key assessment points.

**Figure 2:**
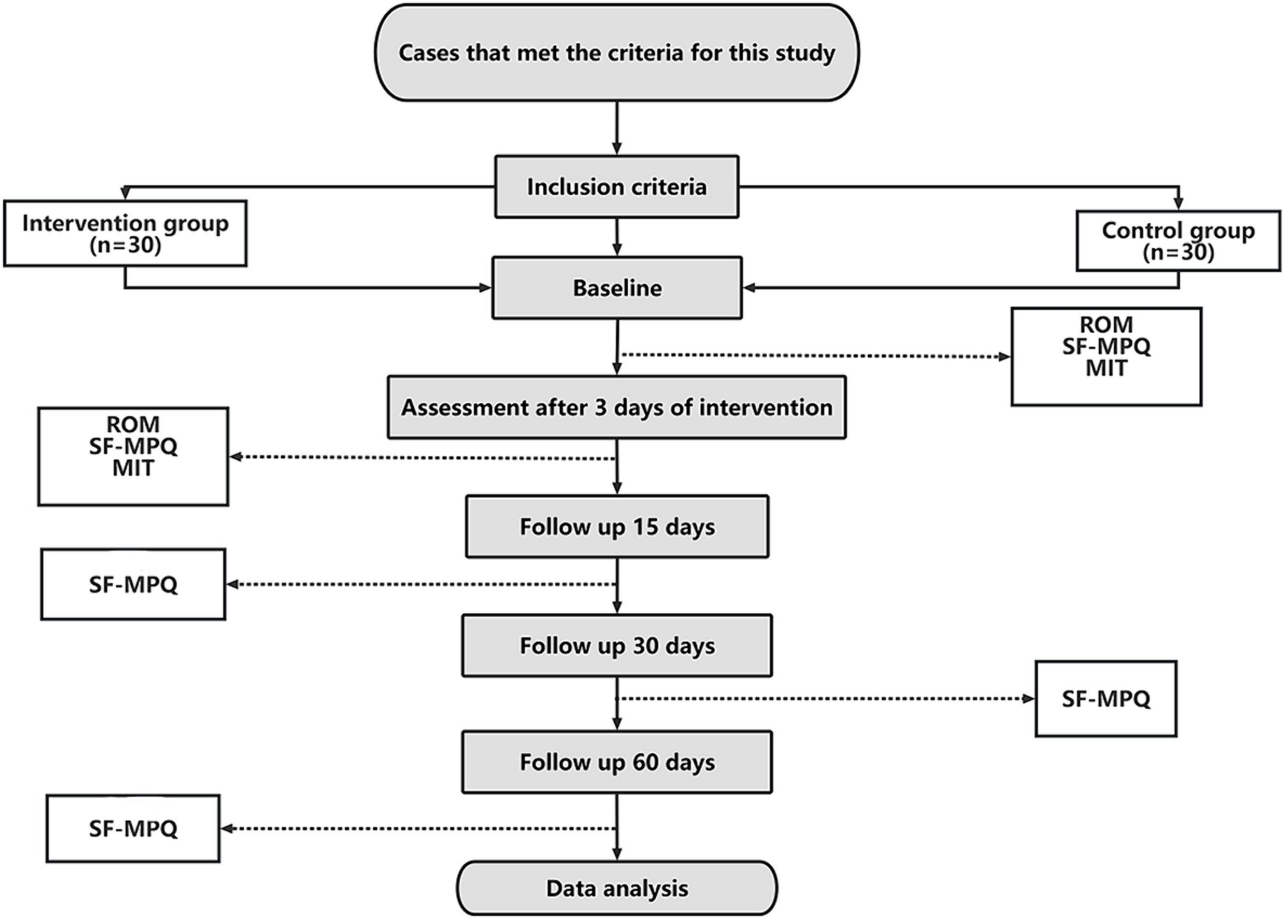
Trial flow chart. This chart provides a visual representation of the trial’s flow, from participant enrollment to the completion of follow-up assessments.

This trial has been registered with the China Clinical Trial Center, bearing the identifier ChiCTR2300076128. It is committed to following the guidelines as set forth by the SPIRIT (Standard Protocol Items: Recommendations for Interventional Trials) statement (27) and adheres to the ethical standards outlined in the Declaration of Helsinki (28) (Figure 1) (see trial flow in Figure 2).

### Participants

#### Inclusion criteria

- Individuals diagnosed with cervicogenic headache (CEH) (1) as per established criteria.
- Participants aged between 18 and 65 years.
- Those willing to participate in the study and who have provided signed informed consent are also included.
- Individuals not currently participating in other clinical trials.
- Only patients with cervicogenic headache who also met a score of ≥5 on the Visual Analogue Scale for Pain (VAS) and had a duration of illness of more than 3 months will be included in the intervention and control groups.

#### Exclusion criteria

- History of head, neck, or shoulder trauma or presence of infectious skin diseases.
- Patients with traumatic headaches, medication-overuse headaches, hypertension-related headaches, or other headache disorders.
- Imaging findings indicating intracranial or extracranial organic lesions, neck fractures, or dislocations.
- Presence of severe cardiac, cerebral, pulmonary, renal, or rheumatoid immune diseases.
- Long-term use of analgesics or sedative-hypnotics that cannot be discontinued for the study duration.
- Pregnant or breastfeeding women.

#### Suspension criteria

- Occurrence or suspicion of a serious adverse event during the trial.
- Confirmation or suspicion of pregnancy during the treatment phase.
- Development of a drug allergy during treatment.

### Recruitment

Recruitment will target patients with CEH at the Tuina Department of Guilin Municipal Hospital of Traditional Chinese Medicine, Guangxi, China. Recruitment strategies include hospital-based poster displays and social media outreach, particularly through platforms like WeChat.

### Patient safety

- The tuina massage will be administered with moderate force to avoid harm, strictly observing contraindications.
- Injections will be performed gently, ensuring slow administration of the medication to avoid entry into the vertebral artery.
- Aseptic techniques will be meticulously observed throughout the injection to maintain patient safety and prevent infection.

### Intervention

Before trial commencement, all participants will be thoroughly informed about the study’s potential risks and benefits. A written informed consent form will be obtained from each participant before randomization.

#### Control group: ultrasound-guided GON and TON injections

The control group will receive injections consisting of a compound betamethasone solution (1 mL of a 7 mg/1 mL concentration, produced by Hangzhou Merck Sharp & Dohme Pharmaceutical Co., Ltd.) diluted with 0.9% sodium chloride solution (4 mL of a 0.9 g/100 mL concentration). The procedural methodology for ultrasound-guided GON and TON injections adheres to the guidelines established by the Chinese Association for the Study of Pain in their “Experts Consensus on Ultrasound-guided Injections for the Treatment of Spinal Pain in China (2020 edition).” Before injection, hair in the occipital region will be trimmed.

#### Ultrasound-guided GON injection procedure

**Figure 3:**
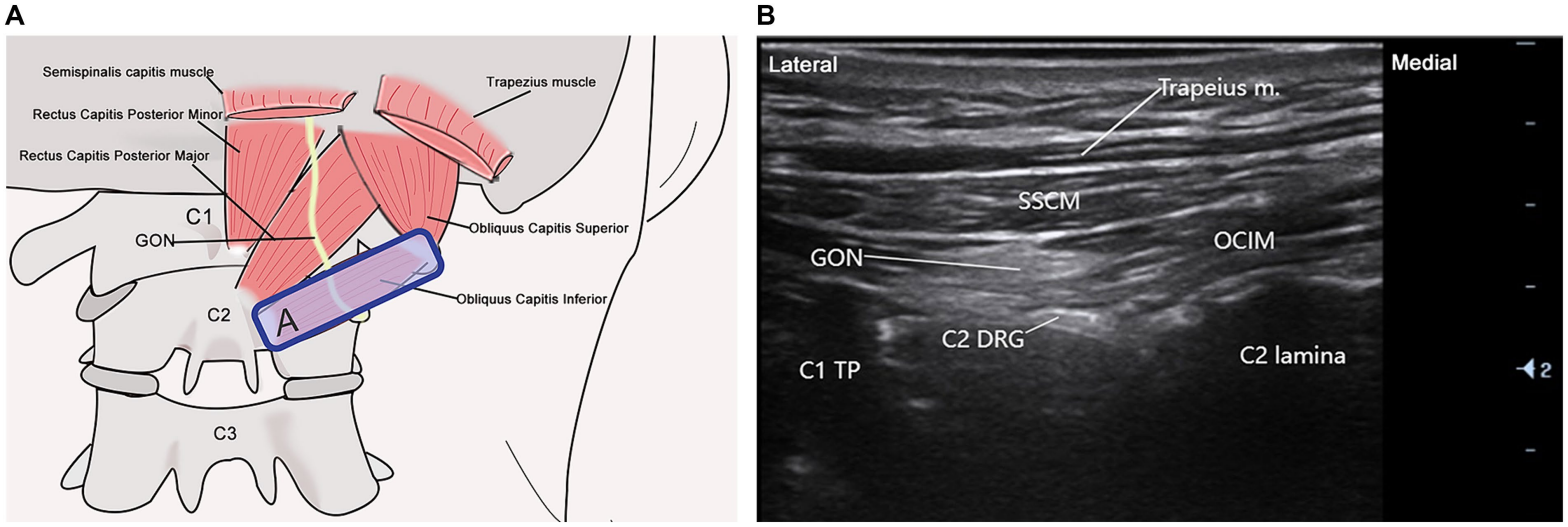
Ultrasound-guided GON scan. **(A)** This illustration illustrates the probe positioning for greater occipital nerve (GON) scanning along the C1 transverse process to the C2 spinous process oblique axis. **(B)** The ultrasonographic image depicts the anatomical relationship between the greater occipital nerve and surrounding structures. Abbreviations include C1 (atlas C1), C2 (axis C2), GON, C1 transverse process (C1 TP), semispinalis capitis muscle (SSCM), obliquus capitis inferior muscle (OCIM), C2 dorsal root ganglion (C2 DRG), and the trapezius muscle. The blue rectangle (A) indicates the probe position.

Patients are prone, with the procedure initiated on the right side for demonstration. The treatment area is first sterilized. A transverse short-axis image of the posterior occipital region is obtained using a high-frequency linear array probe (L14-6Ns, manufactured by Myeri, B-mode, operating frequency 8–12 MHz). The probe is then moved sacro-laterally until an echogenic arc shadow (the spinous process of C1 is not visible in the ultrasound image at this time) is visualized. Moving toward the sacral end reveals the first bifurcated ultrasound image (C2 spinous process). Rotating the probe counterclockwise around the C2 spinous process axis while moving laterally allows for the visualization of the C1 transverse process, resulting in a long-axis scanning image that includes the obliquus capitis inferior muscle (from the C1 transverse process to the C2 spinous process obliquely axial position). The GON is superficially identified in the obliquus capitis inferior muscle in Figure 3. A 5 mL syringe (0.53 mm × 50 mm, 25 G), inserted from the medial edge of the probe and aligned with the ultrasound plane, is used to approach the GON slowly. Upon confirming needle placement via negative aspiration, 2.5 mL of the medication is administered. Following injection, the puncture site is covered with a sterile dressing and localized pressure is applied for 3 min.

#### Ultrasound-guided third occipital nerve (TON) injection procedure

**Figure 4:**
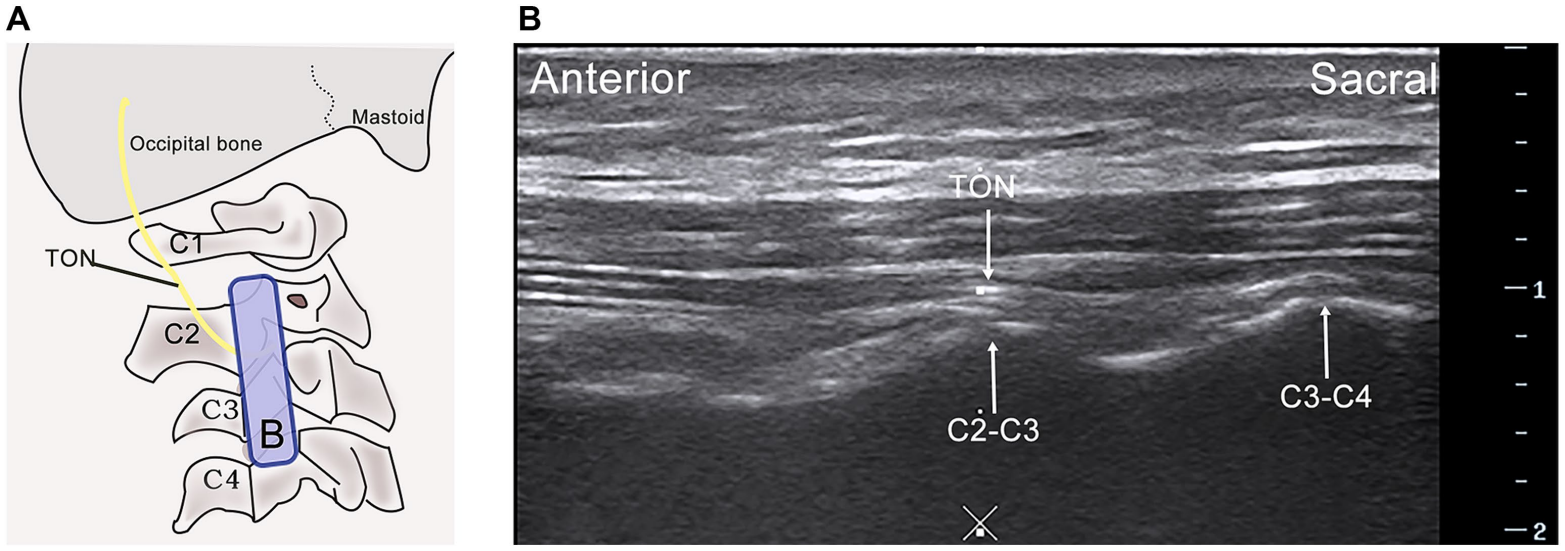
Ultrasound-guided Third Occipital Nerve (TON) scan. **(A)** The probe is placed for the long-axis scan of the C2-C3 articular pillar, illustrating the path of the third occipital nerve. **(B)** Ultrasonographic visualization of the third occipital nerve, highlighting its anatomical relationship with surrounding structures. C2-C3 facet joint (C2-C3), C3-C4 facet joint (C3-C4), C4 vertebra (C4), Midline (×), Probe indication (Blue rectangle B).

Participants are positioned prone to this procedure. The procedure begins by placing the cephalad side of the probe over the mastoid process, aligning it parallel to the cervical spine’s long axis. As the ultrasound probe glides sacralward, the transverse processes of C1 and C2 become visible on the ultrasound display. Continuing the movement sacralward and slightly towards the spinous process, a wave-like acoustic shadow emerges, depicting the articulations between C2-C3 and C3-C4, as illustrated in Figure 4. At this juncture, the TON, traversing over the C2-C3 joint surface, is identified. A 5 mL syringe (0.53 mm × 50 mm, 25 G) is inserted from the probe’s lateral sacral edge with the probe stabilized. The needle is guided in-plane towards the TON, and upon confirming the position via negative aspiration, 2.5 mL of the therapeutic solution is administered. Following injection, the site is covered with a sterile dressing and compressed locally for 3 min to ensure hemostasis.

### Intervention group: ultrasound localized meridian sinew tuina combined with GON and TON injections

#### Treatment protocol

The intervention entails a regimen of daily meridian sinew tuina therapy for three consecutive days, with each session spanning 30 min, starting from the baseline. On the third day, participants receive a single session of ultrasound-guided injections into the greater and third occipital nerves (GON and TON), marking the completion of the treatment course. Each participant undergoes one full course of this combined treatment.

#### Meridian sinew tuina

**Figure 5:**
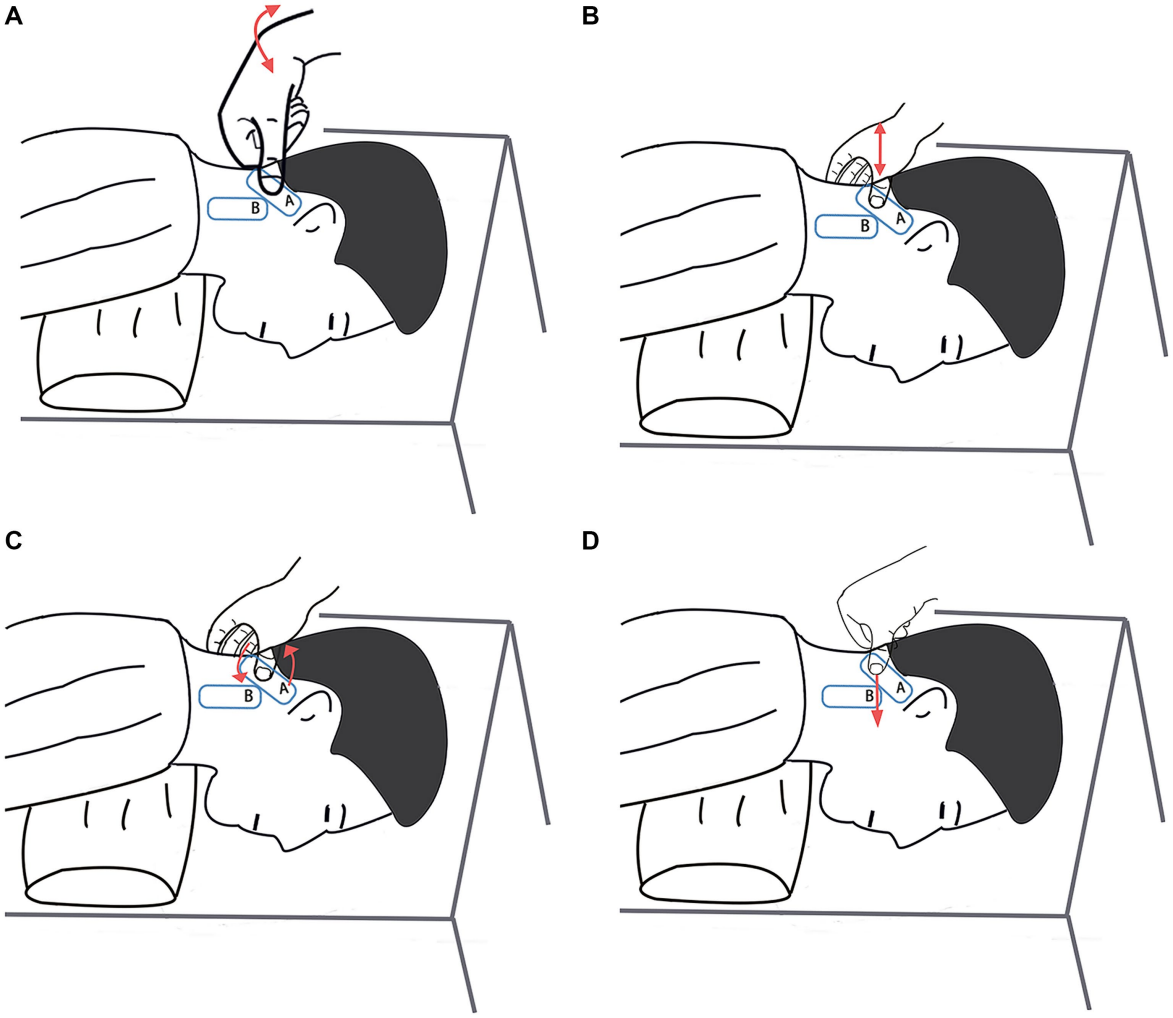
Meridian sinew tuina techniques. **(A)** Dhyana-thumb-pushing (Yi Zhi Chan pushing). **(B)** Plucking. **(C)** Kneading with the Thumb. **(D)** Thumb pressing.

Certified tuina therapists with at least three years of clinical experience in traditional Chinese medicine perform all treatments. These practitioners have undergone rigorous training and assessments in tuina techniques, particularly for this study. Initially, therapists utilize ultrasound to precisely locate the GON and TON on the right side. They then outline the treatment areas “A” and “B” adjacent to the ultrasound probe with a marker, as shown in Figures 3A, 4A. The participant, positioned prone, undergoes specific tuina techniques, including Dhyana-thumb-pushing (Yi Zhi Chan pushing), plucking, kneading with the Thumb, and Thumb pressing in areas “A” and “B” for 20 min, as demonstrated in Figure 5.

Subsequently, the therapist employs palpation to assess the head and neck regions, mapping the superficial to deep layers along the foot’s solar meridian tendons. The examination progresses from gentle to firm pressure, utilizing techniques such as following, pressing, and touching to identify subcutaneous areas of tenderness or nodules. Targeted tuina maneuvers like thumb kneading, Thumb pressing, and lateral plucking are applied to these points, especially along the foot’s solar meridian tendons in the head and neck region. This process also aims to relax muscles such as the trapezius, splenius capitis, semispinalis capitis, rectus capitis posterior major, and Obliquus capitis inferior. The total treatment time for this phase is 10 min.

### Observational indicators

#### Primary observational indicators

##### Short-Form McGill Pain Questionnaire (SF-MPQ)

Pain evaluation is performed using the SF-MPQ, a globally recognized instrument for describing and quantifying pain experiences (29). The SF-MPQ consists of three main components: the Pain Rating Index, which assesses the quality of pain; the Visual Analogue Scale for headache, offering a subjective measure of pain intensity; and the Present Pain Intensity section, which provides a snapshot of the current pain level.

#### Secondary observational indicators

##### Cervical Range of Motion (ROM)

As the American Academy of Orthopedic Surgeons recommended, ROM evaluation is an essential method for quantifying mobility within the cervical joint (30). This measurement involves assessing the extent of movement in various directions from a standardized neutral position, noted as 0°. The established normal ranges of motion are as follows: for flexion and extension, from 0° to 45°; for lateral flexion (both left and right), from 0° to 40°; and for rotation (left and right), from 0° to 70°.

##### Medical infrared thermography

**Figure 6:**
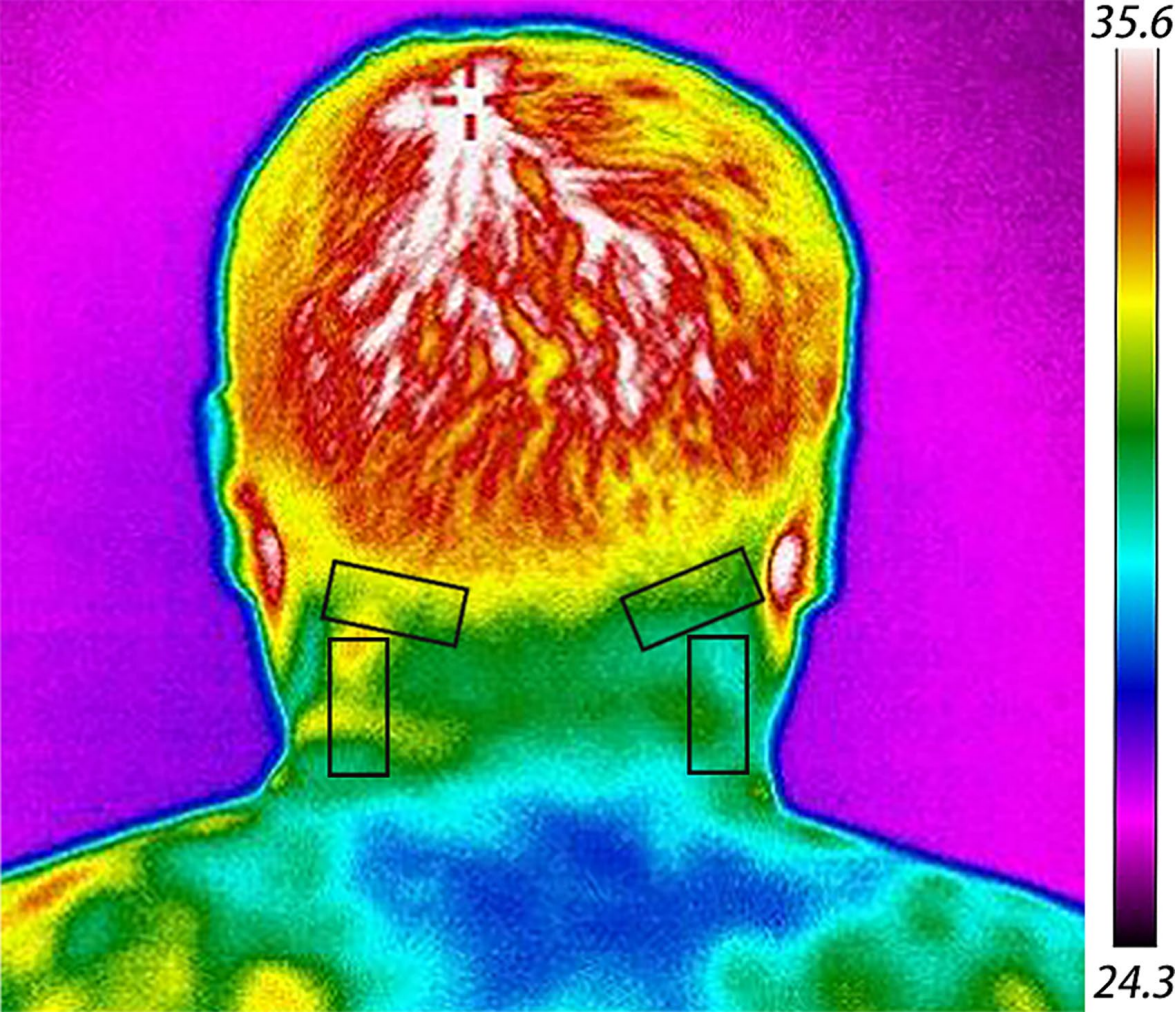
Medical infrared thermography image. The black rectangle delineates the area where data collection occurs.

- *Instrumentation*: The study employs the X640D digital infrared thermography system by Ge Wu You Xin Company. Image analysis is conducted using IRToolPRo software, which provides a comprehensive temperature detection range from 0°C to 300°C with a precision of ±0.3°C. The system boasts a pixel size of 17UM, a frame rate 30 Hz, and operates within the 8 ~ 14UM band. For enhanced visual contrast, the color scale mode is selected.
- *Testing conditions*: The testing environment is precisely controlled to ensure optimal data accuracy. The laboratory temperature is maintained between 20°C and 23°C, with strict avoidance of any direct sunlight or artificial light exposure and minimal indoor air movement (31). Relative humidity is regulated within the 20 to 30% range, creating ideal conditions for thermographic analysis.
- *Operational method*: Participants first acclimate to the laboratory environment by resting quietly for five minutes, allowing skin temperature stabilization. They then stand upright, exposing the head and neck area, and position themselves one meter from the infrared thermography camera. The attending physician adjusts the camera focus to ensure the capture of clear thermal images. Infrared thermal data collection encompasses the highest, lowest, and average temperatures at the skin surface areas corresponding to the bilateral greater occipital nerve and the third occipital nerve, as illustrated in Figure 6.

### Sample size calculation

Derived from preliminary trial results, this study divides patients into two groups via a randomized approach. The primary efficacy endpoint is assessed using the Visual Analog Scale (VAS) scores within the Short-Form McGill Pain Questionnaire (SF-MPQ), with observed mean VAS scores of 2.40 and 3.60, alongside standard deviations of 1.10 and 1.50, for the two groups, respectively. Utilizing G*Power 3.1.9.2 for statistical calculations, with a test significance level (α) set at 0.05 and a power (1-β) of 0.9 for a two-sided test, the sample size requirement for eachup is determined to be 27 participants (32). Factoring in a 10% potential dropout rate, this necessitates 30 participants per group for both the observation and control cohorts.


$$N=2\times {\left[\left(Z_{\alpha /2}+Z_{\beta /2}\right)\times \sigma /\delta \right]}^{2}$$

### Randomization and blinding

The study employs a simple randomization technique, utilizing SPSS 20.0 software for sequence generation, random seeding, and group allocation. Produced and stored in sealed opaque envelopes, randomization cards facilitate assignment anonymity. Eligibility confirmation leads to an envelope opening by researchers external to the treatment, assessment, and statistical analysis processes, ensuring that participant assignment is devoid of bias. Blinding extends to statisticians and data handlers, who remain uninformed of group allocations until post-treatment, enhancing study integrity. Participant treatment is conducted in separate rooms to prevent inter-subject communication.

### Data collection and management

The data collection managers for this study are full-time staff who are clinically trained but not involved in the clinical intervention process, ensuring rigorous verification and accuracy. All findings are strictly derived from original data, with error corrections on case report forms (CRFs) marked without obfuscation. Researchers meticulously document and electronically record patient information, with post-study CRFs securely stored and electronic data fixed against unauthorized modifications. Patient confidentiality is paramount, with participant follow-ups conducted telephonically upon consent.

### Data analysis

The principle of Intention-To-Treat (ITT) analysis and Per-Protocol (PP) analysis will be followed in the process of statistical analysis. Descriptive analysis will be used for the baseline characteristics of the patients in each group, data will be presented as mean or median with standard deviation or interquartile range for continuous variables and as frequency distributions for categorical variables. Two-sample t-test for quantitative data or Chi-square test for qualitative data will be performed as homogeneity test, and analysis of covariance will also be performed if an adjustment is needed for a baseline characteristic. For quantitative data that do not conform to a normal distribution, data are expressed as median (IQR). In all analyses, statistical significance will be accepted as a 2-tailed *p* < 0.05. For the primary outcome, SF-MPQ scores will be assessed by using the linear mixed-effects model with the interaction effects of time and group. Participants who do not complete the study will be treated as having no change from baseline at all times. A Bonferroni correction will be used to account for multiple comparisons. Correlation analysis will be conducted between the difference of medical infrared thermography (MIT) and the other outcome measures by the Pearson correlation analysis. All statistical analyses will be conducted with SPSS software, version 25.0 (SPSS Inc).

## Discussion

Cervicogenic headache (CEH) represents a prevalent headache disorder, posing significant treatment challenges for clinicians. Given its commonality, identifying effective treatment strategies is paramount. Meridian sinew tuina, with its roots stretching back thousands of years within traditional Chinese medicine, has significantly enhanced health and well-being (33). Research suggests that the pain-relieving effects of meridian sinew tuina may stem from a synergy of psychological factors, such as the placebo effect influenced by patient expectations, and physiological mechanisms, including improved local blood circulation, relaxation of tense connective tissues, enhanced local nociception, and the reabsorption of inflammatory mediators (34). These factors collectively contribute to the modulation of central nociception. Hence, it is critical to substantiate the therapeutic Efficacy of meridian sinew tuina in managing CEH through robust evidence (35–37).

In the modern medical landscape, infrared thermography has emerged as a non-invasive, safe, and objective tool for assessing treatment efficacy, diagnosing pain disorders, and investigating traditional Chinese meridians (31). This technology captures the infrared heat radiation emitted by the body, allowing for an intuitive evaluation of temperature variations. Advanced computer-aided color mapping visually represents these temperature differences across the body’s surface, utilizing various colors to highlight temperature anomalies indicative of underlying pathologies (38, 39). In the context of soft tissue inflammation or nerve compression around the head and neck, these disturbances can manifest as either increases or decreases in surface temperature, reflecting alterations in local tissue metabolism and blood circulation. Medical infrared thermography’s capacity to visualize pain sources and objectively measure treatment outcomes offers a novel and objective basis for evaluating CEH treatments (40).

This study heralds several innovative approaches and advantages, primarily in clinical treatment met grohodology. Utilizing ultrasound technology, we precisely identify the anatomical locations of the greater and third occipital nerves (GON and TON), enabling targeted meridian sinew tuina. The integration of meridian sinew tuina with GON and TON injections represents a novel approach that may accelerate recovery in cervicogenic headache (CEH) patients, fostering a synergistic effect between sympathetic nervous system and traditional Chinese therapeutic massage (41). The trial’s design is meticulous, ensuring clarity, transparency, and reproducibility, thereby facilitating the possibility of replication by other researchers.

However, this protocol trial is not without its limitations. Being conducted at a single center, the study’s ability to generalize findings across different populations is restricted. Additionally, the intrinsic characteristics of meridian massage preclude the possibility of blinding practitioners and participants, challenging the implementation of a double-blind randomized controlled trial (RCT). Despite these constraints, the study adheres strictly to separation principles among operators, allocators, and statisticians to mitigate potential biases. The rigorous adherence to treatment protocols aims to yield reliable clinical evidence for CEH management.

In conclusion, our randomized controlled clinical trial is meticulously designed to investigate a safe and effective manual therapy protocol, offering a reference point for the clinical management of CEH. Our aspiration is that the findings from this study will furnish theoretical and practical insights, paving the way for future research in this domain.
